# Preschoolers’ Moderate-to-Vigorous Physical Activity Measured by a Tri-Axial Accelerometer: Compliance with International Guidelines and Different Cut-Points

**DOI:** 10.3390/children11111296

**Published:** 2024-10-26

**Authors:** Aristides M. Machado-Rodrigues, Thales P. Rodrigues da Silva, Larissa L. Mendes, António Stabelini Neto, Helena Nogueira, Daniela Rodrigues, Cristina Padez

**Affiliations:** 1 University of Coimbra, Faculty of Sport Sciences and Physical Education, 3040-248 Coimbra, Portugal; 2 Research Centre for Anthropology and Health, University of Coimbra, 3000-456 Coimbra, Portugal; uc6354@fl.uc.pt (H.N.); drdc@uc.pt (D.R.); cpadez@antrop.uc.pt (C.P.); 3 Nutrition Department, Federal University of Minas Gerais, Belo Horizonte 30130-100, MG, Brazil; thales.philipe@unifesp.br (T.P.R.d.S.); larissaloures@ufmg.br (L.L.M.); 4 Department of Health Science, North Paraná State University, Bandeirantes 86360-000, PR, Brazil; asneto@uenp.edu.br

**Keywords:** active behavior, cut-points, guidelines, preschool, public health

## Abstract

**Background/Objectives**: This study aimed to investigate the effect of the most frequently used accelerometer CoPs on the quantification of active preschoolers by weekday; and to analyze children’s physical activity (PA) quantification using a vertical axis and vector magnitude (VM). **Methods**: A cross-sectional sample of 134 children (70 males) aged 3–5 years was studied. Height, body weight, and BMI were assessed. A tri-axial accelerometer was used for seven consecutive days of MVPA and sedentary behavior (SB). Data were analyzed using the three most used CoPs for active preschooler classification (Johansson, Butte, and Pate). A general linear model with repeated measures examined differences in PA and SB, and the agreements of all CoPs were analyzed using the Kappa index. **Results**: The CoPs adopted by Pate had the highest percentage of children classified as active for the weekdays (73.9%) and weekend (85.6%). The Johansson CoP classified all children as inactive. Furthermore, the prevalence of active boys was significantly higher than their female counterparts based on the Pate and Butte CoPs for the week and weekends. **Conclusions**: The lowest prevalence rates of active children were observed at the weekend based on all accelerometer CoPs, especially among girls. The choice of cut-points significantly affects the times calculated for different movement intensities.

## 1. Introduction

The benefits of higher intensity levels of physical activity (PA), such as the portion of moderate-to-vigorous physical activity (MVPA), on children’s health are widely known, both in terms of metabolic, cardiovascular, and even mental health [1]. However, despite the World Health Organization (WHO) and other international health agencies, together with the governments of several countries worldwide, strongly advocating that children should be physically active, the scientific literature continues to consistently highlight the worrying global prevalence of habitual physical activity (PA) levels in children [2], even at younger ages such as preschoolers. At this initial stage of the lifespan, an accurate assessment of human movement is particularly difficult to attain because of its spontaneous and intermittent features; therefore, it is crucial to achieve reliable measures in research which explore the association between PA and health outcomes. The literature has often reported sex as an important factor that is closely related to 3–5 preschoolers’ active play, as well as objectively measured moderate-to-vigorous physical activity. Portuguese trends have highlighted that most boys and girls meet the current PA guidelines for the total days of assessment [3]. The prevalence rates varied considerably from the week to the weekend in both boys (from 93% at weekdays to 78% at the weekend) and girls (from 78% at weekdays to 68% at the weekend) [4]. Given that sex differences are still observed in preschool children of different geographic backgrounds [5,6] and could have long-term consequences for health disparities between males and females, further research is needed to dissipate potential doubts on whether the observed sex and age differences in PA are present to a similar degree across settings.

In addition, advances in our understanding of preschoolers’ daily PA and sedentary behaviors (SBs) have especially been attributed to technological improvements of motion sensor instruments, which have overcome numerous limitations associated with self-reporting tools [7,8]. Although multi-method approaches are commonly used to assess daily PA patterns in epidemiological studies [9], accelerometers are tools that are widely used to provide objective and valid measures of free-living PA and SB among preschool children. According to the practical and usage procedures of these devices, the time spent at different intensity levels of PA and in SB is strongly dependent on the thresholds applied to the accelerometry “activity counts” to convert these to a biologically meaningful outcome [10,11]. Furthermore, in recent decades, a large range of devices were launched by several manufacturers [e.g., ActiGraph (ActiGraph LLC, Pensacola, FL, USA), Actical (Philips Respironics, Murrysville, PA, USA), ActivPAL (PAL Technologies Ltd., Glasgow, UK)], as were numerous models of accelerometers from within manufacturers, which have made data generalization between studies highly difficult, and, at the same time, quite challenging.

In addition, along with the variety of commercial brands of accelerometers for placement on different body locations (e.g., wrist, hip, and thigh) for behavioral assessments, a complex source of variation which has received less attention is related to the range of validated cut-off points (CoPs) that exist for each accelerometer model. Indeed, discrepancies in CoPs may be due, in part, to the differing criterion methods used during calibration protocols, such as direct observation [12] and indirect [13] or direct calorimetry [14], while children have engaged in standardized treadmill-based or free-living activities, or both. Recent systematic review studies [15,16] revealed different high levels of compliance with the several cut-points to achieve the PA guidelines, with 22 of 35 studies reporting that most children achieved the daily MVPA of the recommendation. Indeed, the previously mentioned findings should be interpreted with caution, as there was considerable variability in the proportion of preschool-aged children meeting the physical activity recommendations across different accelerometer cut-points. For instance, the prevalence of children achieving at least 60 min of daily MVPA in epidemiological studies varied from 0 to 100%. Notably, the Pate cut-points [13] were the most consistently used CoPs to evaluate the compliance of both individual and the overall WHO recommendations in preschooler studies. Pooled estimates based on this cut-point also demonstrated that 60% of children (C) achieved the overall PA recommendation, and 90% achieved the MVPA aspect of the recommendation [16]. Therefore, the methodological issues and controversial interpretations, as well as the use of several age groups from different sociocultural contexts in recent studies, make the comparison of estimates between studies complex and justifies further research.

In the context of the preceding trends and taking into account the literature [17,18], which has clearly highlighted that for public health and policy makers’ interpretations of PA recommendations, it is more effective to use a straightforward message, such as a specific duration of time (i.e., 60 min), rather than a recommendation expressed in counts per minute (cpm) from accelerometry data. Therefore, the purpose of this study was to determine the assessment discrepancies of SB, light PA, and MVPA in the application of preschool children cut-point thresholds provided by Pate and colleagues [12], Johansson and colleagues [19], and Butte and colleagues [14] using the ActiGraph GT3X+ accelerometer. It was hypothesized that the percentage of active children would be different according to sex and specific accelerometry CoPs, since these both come from different calibration studies with different methodological approaches, sample sizes, and sociocultural contexts.

## 2. Materials and Methods

### 2.1. Participants

The data were obtained from an epidemiological large-scale project entitled “The Inequalities in Childhood Obesity: the impact of the socioeconomic crisis in Portugal”, a random cross-sectional survey conducted in school children from the Portuguese midlands. A total of 8430 school-aged children aged from 3 to 10 years old (50.8% male) were recruited from 118 schools, public and private, in the cities of Porto, Coimbra, and Lisbon [20]. The present study is a specific part of the afore-mentioned project, which included preschool children from the randomly selected schools in the midlands. The final sample of the present study included all children aged 3–5 years [the mean age of girls was 4.45 (±0.9) years, and for boys, it was 4.55 (±0.8) years] whose parents authorized their participation in all stages of the project; children with missing information on accelerometry (see all criteria below at PA measurements) were also excluded from the sample. Among the 134 children (74 males) who participated in the current study, 20% of females and 12% of males were overweight or obese. Fifty-five percent of those participants had a father with a college or university degree (highest educational level), and 11% had a father with the lowest education level (e.g., nine compulsory years). Furthermore, 76% of the children had mothers with the highest education level, and 5% had mothers with the lowest education level. Ethical approval for the project was given by the *Direcção-Geral de Inovação e Desenvolvimento Curricular* (Study Registration NO. 0565500003/DGIDC; 28 October 2016), which requires anonymity and non-transmissibility of data, according to the guidelines laid down in the Declaration of Helsinki of 1975, revised in 2013. Moreover, prior to data collection, informed written assent was obtained from parents or guardians.

### 2.2. Anthropometric Measures

Body weight and height were objectively measured by trained researchers while the children were in a standing position without shoes and with minimal clothing, according to established protocols. All children were measured for body weight with a digital scale (Seca, Hamburg, Germany) and height with a portable stadiometer (Seca 225, Hamburg, Germany). The Body Mass Index (BMI; kg/m^2^) was subsequently calculated and categorized using age- and sex-adjusted CoPs [21]. The sample was divided into two body weight status groups, normal body weight and overweight/obese.

### 2.3. Physical Activity (PA) and Sedentary Behavior (SB) Measurement

PA and SB were objectively measured for seven consecutive days using a wGT3X-BT Actigraph accelerometer (ActiGraph LLC, Pensacola, FL, USA). The tri-axial accelerometer was placed over the hip using an elastic belt above the right anterior superior iliac spine. The acceleration signal was filtered and digitized, and the magnitude was aggregated over a user-defined period of time (an epoch interval), which was set to 5 s, similarly to other studies of preschool children; this epoch has been used as the more accurate for evaluating the spontaneous and intermittent activities of preschoolers [22].

Accelerometer data were electronically downloaded using the ActiLife 6 software. Children who did not complete a minimum of 10 h of daily accelerometer data after removing sequences of 20 or more consecutive zero counts [23,24] were immediately excluded from subsequent analyses. The accelerometer output was interpreted using intensity-based cut-points, which categorize activity counts as sedentary, light, moderate, or vigorous PA. The total amount of daily sedentary behavior, light PA, and MVPA were expressed in minutes/day, and it was calculated using a specific pediatric cut-point for preschool-aged children proposed by Pate and colleagues [12], Butte and co-workers [14], as well as Johansson and colleagues [19]: single axis cut-points were <800 counts per minute (cpm) [12], <239 cpm [14], and <580 cpm [19] for SB; and ≥1680 cpm [12], ≥2120 cpm [14], and ≥3480 cpm [19] for MVPA. Furthermore, the Vector Magnitude cut-points were established as follows: <820 cpm [14] and <2136 cpm [19] for SB; and ≥1908 cpm [14] and ≥6144 cpm [19] for MVPA. Participants were classified as active if they accumulated at least 60 min of MVPA per day (≥60 min/d of MVPA) and inactive if they did not reach these recommended values (<60 min/d of MVPA), according to several epidemiological studies [25].

### 2.4. Statistical Analysis

Prior to analysis, tests for normality were conducted on the indicators of body size and sedentary time, light physical activity, and MVPA. Data are expressed as mean and 95% confidence intervals (95% CI).

A repeated-measures *t*-test was used to compare two within-subject observations of a continuous variable. The a priori criteria were as follows: effect size 0.5; error (alpha) 0.05; and power 95%, requiring at least 54 subjects (critical T of 2.00). A general linear model with repeated measures examined the differences in time spent at the different intensity levels of PA and SB for all cut-points. Bonferroni corrections were used to adjust the 95% CI.

The agreements of the proportions of children who were physically active (≥60 min/day of MVPA) on weekdays and at the weekend were analyzed using the Kappa index. The agreement of all CoPs was analyzed using the Kappa index. The parameters used as reference points to classify the Kappa index and the ICC were excellent agreement (0.80 to 1.00), substantial (0.60 to 0.79), moderate (0.40 to 0.59), reasonable (0.20 to 0.39), weak (0 to 0.19), and no agreement (≤0) [26].

Statistical analysis was conducted using the Statistical Package for the Social Sciences version 19.0 (IBM Corp., Armonk, NY, USA). Statistical significance was set at 5%.

## 3. Results

Findings of the present study revealed that there were no significant differences between males and females for BMI (boys: 15.81; girls: 16.06). About 80.0% of the girls were categorized as normal body weight, 18.3% as overweight, and 1.7% as obese; the corresponding values for the boys were 87.8%, 8.1%, and 4.1% for normal body weight, overweight, and obese, respectively.

Table 1 shows the daily total minutes spent on the different portions of PA and SB on the weekdays by sex. Among the boys, after Bonferroni correction, the results revealed significant differences for sedentary activity time (Pate = 506.35 min/day; Butte = 420.87 min/day; and Johansson = 526.40 min/day), light physical activity (Pate = 57.94 min/day; Butte = 159.64 min/day; and Johansson = 112.58 min/day), and MVPA (Pate = 86.09 min/day; Butte = 69.86 min/day; and Johansson = 11.24 min/day) for all CoPs. Regarding the girls, for sedentary activity time, similar means were observed between the Pate (539.96 min/day) and Johansson (561.98 min/day) CoPs, i.e., no significant differences were observed; however, there were significant differences between the Butte CoP (449.27 min/day) and the other two criteria. Furthermore, the results revealed significant differences for light physical activity (Pate = 58.74 min/day; Butte = 164.76 min/day; and Johansson = 105.37 min/day) and MVPA (Pate = 77.79 min/day; Butte = 62.47 min/day; and Johansson = 9.55 min/day) for all CoPs (Table 1).

Similar results were observed at the weekend (Table 2). Thus, among boys, the results revealed significant differences for sedentary activity time (Pate = 606.54 min/day; Butte = 505.74 min/day; and Johansson = 627.47 min/day), light physical activity (Pate = 63.32 min/day; Butte = 180.13 min/day; and Johansson = 121.14 min/day), and MVPA (Pate = 90.87 min/day; Butte = 74.89 min/day; and Johansson = 12.24 min/day) for all CoPs.

Among the girls, for sedentary activity time, similar means were observed between the Pate (633.28 min/day) and Johansson (656.35 min/day) CoPs, i.e., no significant difference, but there were statistically significant differences between these and the Butte CoP (531.51 min/day).

Similar results were observed for the total of all assessed days (Table 3).

Participants were classified as active or inactive according to international PA guidelines (i.e., ≥60 min of MVPA) and each CoPs, and the results are presented in Table 4. The results revealed that the CoPs adopted by Pate had the highest percentage of children classified as active (87.3%) for the total assessed days: 73.9% for the weekdays and 85.6% at the weekend. The Johansson CoPs classified all children of both sexes as inactive (i.e., <60 min of MVPA for all analyzed periods). When observing the differences in the proportions of boys and girls who were classified as active (i.e., ≥60 min of MVPA), significantly higher proportions of boys were classified as active children than girls with the Pate and Butte CoPs (Table 4) at the weekend and for all assessed days.

Table 5 presents the Kappa index of the proportion of male children who were classified according to the recommendation of active participants for each CoPs. Moderate agreement was observed for the Pate and Butte CoPs on weekdays (Kappa = 0.576) and reasonable for both the total of all assessed days (Kappa = 0.265) and the weekend (Kappa = 0.367), with significant differences. No agreement was observed in the comparison of the Johansson CoPs.

Among females, substantial agreement was found for the Pate and Butte CoPs on weekdays (Kappa = 0.692). For the total of all assessed days (Kappa = 0.471) and the weekend (Kappa = 0.456), the agreement for Pate and Butte was moderate (*p* < 0.01). No agreement was observed in the comparison of the Johansson CoPs (Table 6).

Finally, a comparison between the counts/min provided by the uniaxial vector (y) and the magnitude vector (MV) was performed for the Pate CoPs (e.g., the most used accelerometer CoPs for data analysis among preschoolers). The findings revealed that the mean of counts per minute (cpm) of the magnitude vector was significantly higher than those provide by the uniaxial vector (y-axis) for all assessed days (y: 535 cpm versus MV: 1179 cpm), for weekdays (y: 534 cpm versus MV: 1162 cpm), and for weekend days (y: 537 cpm versus MV: 1186 cpm). Similar results were observed for males and females, separately.

## 4. Discussion

The World Health Organization recommends active behaviors to manage the concerning worldwide prevalence of childhood obesity, which is still growing in different geographic contexts in the south of Europe, such as in countries like Portugal [27]. Therefore, meaningful conclusions about the prevalence of PA and the outcomes of interventions, based on valid, reliable, and feasible measures, are increasingly required, particularly in less-studied age groups of preschoolers. Thus, the present study aimed to determine the discrepancies of measurements of time spent in SB, light-intensity PA, and MVPA upon the application of cut-point thresholds developed specifically for preschool children, and it revealed that the analysis approach of accelerometer-derived data has a huge impact on the MVPA outcome. For example, the CoPs adopted by Pate had the highest percentage of children who were classified as active (87.3%) for the total of all assessed days: 73.9% for the weekdays and 85.6% at the weekend. In addition, the Johansson CoPs classified all children of both sexes as inactive (i.e., <60 min of MVPA for all analyzed periods). Similar discrepancies between classifications of children’s activity levels were observed among preschool children in Switzerland [28], the UK [29], Finland [15], and Spain [30].

Despite accelerometers being viewed as the best-practice methodology for PA assessments in children aged 0 to 5 years [11,16], providing the most valid estimates, caution is needed for interpretations and data generalization, since the analytical design of studies, as well as methodological procedures (i.e., the placement of the device; epoch selection; assessed days; and, particularly, CoPs used), are quite diverse across studies of the same age range. The present study revealed that the time spent at different PA intensities differs substantially depending on the applied cut-point. Of note, the MVPA cut-points were derived from different samples with divergent biological and cultural characteristics. For example, Pate and colleagues [12] included urban U.S. children who were older than the Swedish children from rural areas included in the study by Johansson and colleagues [19]. Furthermore, the literature generally indicates that MVPA is more likely to be misclassified as light physical activities; in fact, this is clearly evident when preschool children engage in activities that require higher levels of energy expenditure but relatively little movement, such as climbing on fixed equipment in the playground [31]. On the other hand, activities such as walking are frequently misclassified as light physical activity, because under true free-living conditions, preschoolers often walk in a slow and meandering manner that is consistent with the concept of “pottering around” [31]. It is important to highlight that the development of these PA recommendations has been based mainly on self-reported PA data [32] and, as a result, these rates should be interpreted with caution. Nevertheless, more research is needed to provide information on health-related PA based on objective methods between studies.

Of interest, the time spent being sedentary was quite similar when assessed by the Pate and Butte CoPs, but they revealed substantial differences in comparison with the Johansson CoPs among both males and females. The difference between the SB CoPs is perhaps due to a discrepancy in the definition of SB adopted by the different observational studies. Specifically, in the study by Johansson and colleagues, SB was defined as “stationary with no movement and stationary with movement of the limbs”, resulting in higher CoPs [19] than those of Pate and colleagues [12] or Butte and co-workers [14], who did not include limb movement. Indeed, previous research has also shown that SBs are more likely to be misclassified as light physical activity if they are performed with significant upper body movement [33]. On the other hand, it should be clearly highlighted that differences in the validation protocols are not just based on the conceptual approaches and criterion measures, which are diverse across various studies [12,13,14,19], but they are also related with the settings in which PA is assessed (i.e., free-living PA assessment versus laboratory settings). As a result, there are a large range of CoPs that generate disparity in PA estimates, leading to lack of comparability. Indeed, the specificity of validation protocols also plays an important role in the potential generalizability of results. For example, most of the studies reported the general features of included children, such as chronological age and sex. However, few studies reported other key attributes that help determine the generalizability of the results to the wider population, including ethnic origin and socioeconomic status [11]. Therefore, there is a consensus that there is limited evidence indicating that the results from individual studies can be generalized to other populations.

Interestingly, the present study found similar sex-related trends in the agreement between CoPs; thus, for both boys and girls, no agreement was observed in the comparison of the Johansson CoPs with the others (i.e., Pate and Butte). These last CoPs have shown similar trends in their discrimination of PA and SB levels between boys and girls. Indeed, the sex differences based on lifestyle between sexes are especially evident at the transition from childhood to adolescence. Hence, at these ages of preschool education, as children are very dependent on their daily routine being set by an adult (i.e., teachers or parents), the patterns of PA [34] and SB [35] between boys and girls are very similar. Thus, these combined studies showed that in both sedentary, light, and MVPA, the differences in the activity distribution for girls and boys were marginal, with small disparities in the variation for either measure or for the total volumes. Indeed, the time spent at these intensities accounted for most of the waking hours, indicating that differences in overall activity (i.e., cpm) are driven by a small sub-set of daily behaviors at the moderate-to-vigorous thresholds. Nonetheless, among European nations, while almost all countries show similar sex differences for children, Portuguese children did not reveal as much of a sex-related difference in total activity, expressed by cpm, as other countries have [36].

Another source of variation is related to the contrast of using different axes for assessing the intensity levels of the PA of preschool children. For example, when using the vector magnitude (VM), in comparison with the vertical axis, preschoolers were classified as substantially more active; in addition, they presented less SB and more light PA and MVPA [28]. These findings are in line with our study revealing significant differences in activity counts between individual axes and the vector magnitude (i.e., a three-dimensional system). The combined results may not be surprising once the VM cut-points take into account the vertical axis, as well as the longitudinal and lateral axes. Generally, using data from the VM to categorize the activity intensity provided better results (higher agreement between the accelerometry and comparison measure) than the vertical axis (y-axis) [19,37], especially for activity type classification (i.e., running, walking, climbing up/down, crawling, riding on a ride or toy, standing, sitting, being in a stroller, or being carried) [38]. However, according to recent studies, when the main purpose of the study is just to differentiate intensity levels of PA, CoPs using hip-based vector magnitude data do not result in more precise classifications of SB, light PA, and MVPA compared to classifications using hip-based vertical axis data [39,40]. The lack of adequate CoPs may be explained by the highly variable and omnidirectional activity pattern of preschoolers and emphasizes the need for more advanced analysis approaches, as well as accompanying user-friendly tools for the application of such approaches in practice. Therefore, supported by previous research [40], future studies could capture the various intensities of preschoolers’ PA using the whole intensity spectrum, e.g., by applying the multivariate PA signature.

To the best of our knowledge, this study is one of the first studies to demonstrate the effect of different CoPs assessed by tri-axial accelerometry on the recommended guidelines for MVPA in European preschoolers. The major strengths of this study are the specific age features of the population sample in the context of its demographic characteristics as Southern European participants with high rates of overweight and obesity. Furthermore, the present study analyzing tri-axial accelerometry data for three time periods, according to three different cut-off values that were not included in other studies, and in a sample of children aged 3–5 years for seven consecutive days—which are always difficult to evaluate and research—is extremely relevant to epidemiology and public health studies of pediatric populations. However, the limitations of the present study should also be recognized; firstly, this study has a cross-sectional design and, therefore, it is not possible to infer causal relationships. Furthermore, other social and cultural factors which were not considered in the present study might impact on the afore-mentioned analytical approach; finally, because there is a potential recruitment bias due to the involvement of only volunteering schools, the CoPs found to be the most appropriate need to be tested before any generalization to the broader population. Although it is desirable to provide recommendations for tri-axial accelerometry data analysis for preschoolers, the present study just aimed to analyze the magnitude of differences in the quantification of active children according to the international MVPA guidelines. Indeed, the choice of accelerometer CoPs had a substantial impact on the measured time spent at different PA intensities [8,31]. Both the CoPs and the choice of axis must be considered when comparing different studies and may explain part of the differences in the observed PA and time spent sedentary. Thus, since the cut-point selection applied to data from preschoolers significantly affects the times calculated for different movement intensities, comparisons of movement intensities by accelerometry should not be made across studies using different sets of CoPs. In this way, we will be accurately classifying inactive children, and, thus, we will make a decisive contribution to making more effective public health intervention policies and programs.

## 5. Conclusions

The lowest prevalence rates of active preschool children were observed at the weekend by the most frequently used accelerometer CoPs (i.e., Pate and colleagues), especially among girls. The choice of CoPs applied to data from preschoolers significantly affects the times calculated for different movement intensities, which in turn impacts the proportion of 3–5-year-old children meeting the PA guidelines. Thus, the highly variable and omnidirectional activity pattern of preschoolers may explain the lack of adequate CoPs, and consequently, comparisons of movement intensities should not be made across studies using different sets of CoPs. Further research is needed to confirm and extend values for directly measured cadences, associated speeds, and MET values in younger pediatric people.

## Figures and Tables

**Table 1:** Total daily minutes spent on the different portions of Physical Activity (PA) and Sedentary Behaviors (SBs) for weekdays, separated by sex.

		Males (n = 74)			Females (n = 60)		
		Mean (SE)	CI (95%)	Greenhouse–Geisser	Mean (SE)	CI (95%)	Greenhouse–Geisser
Sedentary time [min/day]	Pate	506.35 (17.26) ^A^	471.95–540.75	0.552 **	539.96 (22.86) ^A^	494.22–585.71	0.526 **
	Butte	420.87 (14.96) ^B^	391.05–450.69		449.27 (21.17) ^B^	406.90–491.65	
	Johansson	526.40 (17.92) ^C^	490.68–562.12		561.58 (23.46) ^A^	514.63–608.53	
Light PA [min/day]	Pate	57.94 (2.28) ^A^	53.38–62.49	0.741 **	58.74 (2.65) ^A^	53.43–64.04	0.804 **
	Butte	159.64 (6.33) ^B^	147.01–172.27		164.76 (7.14) ^B^	150.46–179.06	
	Johansson	112.58 (4.65) ^C^	103.30–121.86		105.37 (5.47) ^C^	94.41–116.33	
MVPA [min/day]	Pate	86.09 (4.05) ^A^	78.01–94.17	0.555	77.79 (4.30) ^A^	69.19–86.40	0.538
	Butte	69.86 (3.33) ^B^	63.21–76.51		62.47 (3.67) ^B^	55.10–69.83	
	Johansson	11.39 (1.04) ^C^	9.30–13.48		9.55 (0.68) ^C^	8.18–10.91	

** *p* < 0.001; PA (Physical Activity); MVPA (Moderate-to-Vigorous Physical Activity); Standard Error (SE); confidence interval (CI); equal letters mean similarity between the means, that is, there is no statistically significant difference based on Bonferroni correction.

**Table 2:** Total daily minutes spent on the different portions of Physical Activity (PA) and Sedentary Behaviors (SBs) for weekend days, separated by sex.

		Males (n = 74)			Females (n = 60)		
		Mean (SE)	CI (95%)	Greenhouse–Geisser	Mean (SE)	CI (95%)	Greenhouse–Geisser
Sedentary time [min/day]	Pate	606.54 (10.35) ^A^	585.90–627.19	0.574 **	633.28 (12.28) ^A^	608.70–657.87	0.569 **
	Butte	505.74 (9.85) ^B^	486.11–525.38		531.51 (12.63) ^B^	506.23–556.79	
	Johansson	627.47 (10.52) ^C^	606.50–648.44		656.35 (12.40) ^A^	631.52–681.17	
Light PA [min/day]	Pate	63.32 (1.32) ^A^	60.68–65.97	0.762 **	61.47 (1.90) ^A^	57.66–65.28	0.727 **
	Butte	180.13 (4.24) ^B^	171.66–188.59		179.20 (5.33) ^B^	16853–189.87	
	Johansson	121.14 (2.90) ^C^	115.35–126.92		106.02 (3.54) ^C^	98.92–113,13	
MVPA [min/day]	Pate	90.87 (2.53) ^A^	85.82–95.93	0.708 **	77.60 (2.79) ^A^	72.00–83.20	0.585 **
	Butte	74.89 (2.29) ^B^	70.32–79.46		61.64 (2.32) ^B^	56.99–66.29	
	Johansson	12.24 (0.59) ^C^	11.06–13.42		9.98 (0.54) ^C^	8.89–11.08	

** *p* < 0.001; PA (Physical Activity); MVPA (Moderate-to-Vigorous Physical Activity); Standard Error (SE); confidence interval (CI); equal letters mean similarity between the means, that is, there is no statistically significant difference based on Bonferroni correction.

**Table 3:** Total daily minutes spent on the different portions of Physical Activity (PA) and Sedentary Behaviors (SBs) for the seven assessed days, separated by sex.

		Males (n = 74)			Females (n = 60)		
		Mean (SE)	CI (95%)	Greenhouse–Geisser	Mean (SE)	CI (95%)	Greenhouse–Geisser
Sedentary time [min/day]	Pate	577.92 (9.51) ^A^	558.96–596.88	0.579 **	606.62 (13.31) ^A^	579.99–633.25	0.541 **
	Butte	481.50 (8.99) ^B^	463.56–499.43		508.01 (13.45) ^B^	481.10–534.93	
	Johansson	598.59 (9.76) ^C^	579.13–618.05		629.27 (13.53) ^A^	602.19–656.35	
Light activity [min/day]	Pate	61.78 (1.26) ^A^	59.26–64.30	0.735 **	60.69 (1.83) ^A^	57.01–64.37	0.753 **
	Butte	174.27 (4.01) ^B^	166.26–182.28		175.07 (5.08) ^B^	164.90–185.25	
	Johansson	118.69 (2.66) ^C^	113.93–124.00		105.84 (3.56) ^C^	98.71–112.96	
MVPA [min/day]	Pate	89.51 (2.36) ^A^	84.79–94.23	0.713	77.66 (2.78) ^A^	72.08–83.23	0.582
	Butte	73.45 (2.06) ^B^	69.34–77.56		61.88 (2.35) ^B^	57.16–66.59	
	Johansson	12.00 (0.58) ^C^	10.83–13.17		9.86 (0.47) ^C^	8.91–10.80	

** *p* < 0.001; PA (Physical Activity); MVPA (Moderate-to-Vigorous Physical Activity); Standard Error (SE); confidence interval (CI); equal letters mean similarity between the means, that is, there is no statistically significant difference based on Bonferroni correction.

**Table 4:** Classification of active children (> 60 min/day of MVPA) by different cut-off points on weekdays, at the weekday, and for the total of all assessed days, separated by sex.

% of Active Children by Cut-Off Points for Total Sample and for Males and Females Separately							
	Total		Sex				
			Males		Females		*p*-Value
	≥60 min	<60 min	≥60 min	<60 min	≥60 min	<60 min	
Weekend days							
Pate	116 (85.57)	18 (13.43)	69 (93.24)	5 (6.76)	47 (78.33)	13 (21.67)	0.012
Butte	87 (64.93)	47 (35.07)	56 (75.68)	18 (24.32)	31 (51.67)	29 (48.33)	0.004
Johansson	0	134 (100.00)	-	74 (100)	-	60 (100)	-
Weekdays							
Pate	99 (73.88)	35 (26.12)	58 (78.38)	16 (21.62)	41 (68.33)	19 (31.67)	0.188
Butte	76 (56.72)	58 (43.28)	44 (59.46)	30 (40.54)	32 (53.33)	28 (46.67)	0.477
Johansson	0	134 (100)	-	74 (100)	-	60 (100)	-
Total days (seven days)							
Pate	117 (87.31)	17 (12.69)	71 (95.95)	3 (4.05)	46 (76.67)	14 (23.33)	0.001
Butte	91 (67.91)	43 (32.09)	58 (78.38)	16 (21.62)	33 (55.00)	27 (45.00)	0.004
Johansson	-	134 (100)	-	74 (100)	-	60 (100)	-

MVPA (Moderate-to-Vigorous Physical Activity).

**Table 5:** Kappa index values of the classification of male children according to the recommendation for active individuals for each cut-off point.

	Pate	Butte
	Agreement (Kappa)	Agreement (Kappa)
Total days (7 days)		
Pate	-	-
Butte	82.42% (0.265) **	-
Johansson	4.05% (0.000)	21.62% (0.000)
Weekdays		
Pate	-	-
Butte	81.08% (0.576) **	-
Johansson	21.62% (0.000)	40.54% (0.000)
Weekend days		
Pate	-	-
Butte	82.43% (0.367) **	-
Johansson	6.76% (0.000)	24.32% (0.000)

** *p* < 0.001.

**Table 6:** Kappa index values of the classification of female children according to the recommendations for active individuals for each cut-off point.

	Pate	Butte
	Agreement (Kappa)	Agreement (Kappa)
Total days (7 days)		
Pate	-	-
Butte	75.00% (0.471) **	-
Johansson	23.33% (0.000)	45.00% (0.000)
Weekdays		
Pate	-	-
Butte	85.00% (0.692) **	-
Johansson	31.67% (0.000)	46.67% (0.000)
Weekend days		
Pate	-	-
Butte	73.33% (0.456) **	-
Johansson	21.67% (0.000)	48.33% (0.000)

** *p* < 0.001.

## Data Availability

The data presented in this study are available on request from the corresponding author due to privacy and ethical restrictions.
